# Nocardia cyriacigeorgica brain abscess in a patient on low dose steroids: a case report and review of the literature

**DOI:** 10.1186/s12879-022-07612-y

**Published:** 2022-07-21

**Authors:** Segev Gabay, Michal Yakubovsky, Ronen Ben-Ami, Rachel Grossman

**Affiliations:** 1 Department of Neurosurgery, Tel Aviv Sourasky Medical Center, Tel-Aviv University, 6 Weizman Street, 6423906 Tel Aviv, Israel; 2 Departments of Infectious Diseases and Infection Control, Tel Aviv Sourasky Medical Center, Tel-Aviv University, Tel Aviv, Israel; 3 Department of Infectious Diseases and Infection Control, Tel Aviv Sourasky Medical Center, 6 Weizman Street, 6423906 Tel-Aviv, Israel

**Keywords:** N. cyriacigeorgica, Brain abscess, Immunocompetent, Case report

## Abstract

**Background:**

Nocardia cyriacigeorgica was first described in 2001. It is an emerging pathogen that mainly affects immunocompromised patients. A brain abscess caused by N. cyriacigeorgica has been reported only in immunocompromised hosts. We present a rare case of brain abscess caused by N. cyriacigeorgica in an adult male receiving low dose steroids.

**Case presentation:**

A 75-year-old male weekend gardener without an immunocompromising condition presented with neurological complaints that were initially attributed to an ischemic stroke. Due to the unusual presentation and rapid progression, his condition was thought to be caused by a cerebral space-occupying lesion. He underwent an emergent right-sided parietal craniotomy and the histopathological report of the specimen was an abscess caused by N. cyriacigeorgica. The patient received appropriate antibiotic treatment and completely recovered without sequelae.

**Conclusions:**

Nocardia species are a rare cause of brain abscess in immunocompetent patients. Their clinical presentation can mimic other more common cerebral diseases, such as brain tumors (primary and secondary) and stroke. The possibility of an abscess caused by N. cyriacigeorgica should also be considered in the differential diagnosis in an immunocompetent patient.

## Background

Nocardia cyriacigeorgica is an emerging pathogen [1], first described in 2001 and since then has been reported in many countries worldwide. Most clinical cases involved immunocompromised patients [2, 3], including several case reports describing brain abscesses [6–8]. Mainly cutaneous infections were observed in immunocompetent hosts [4] and invasive infections are rare [9]. Nocardial brain abscesses are believed to be a sequela of hematogenous spread of lung infection [5]. To the best of our knowledge, a N. cyriacigeorgica brain abscess has not been reported in immunocompetent persons. Here, we describe an unusual case of a N. cyriacigeorgica brain abscess in an adult male on long term, low dose steroids.

## Case presentation

**Fig. 1:**
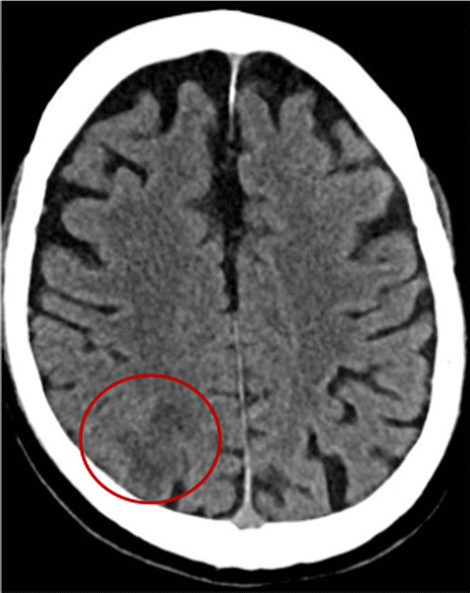
Non-contrast computed tomography (CT) scan performed in the emergency department showing a right parietal hypodense area

We describe the case of a 75-year-old right hand-dominant male who was admitted to hospital with acute confusion and left-hand apraxia. His medical history included past smoking (35 pack-years, quit 30 years ago), ischemic heart disease, hypertension, chronic kidney disease (baseline creatinine levels of 1.5 mg/DL), dyslipidemia, well-controlled mild asthma, hypothyroidism, and locally invasive prostate carcinoma with good systemic control and complete remission under hormonal therapy and 5 mg prednisone per day during the last 18 months. Notably, the patient did not suffer from diabetes mellitus, however, his long term use of low dose steroid does possess a risk factor for immunosuppression. He had traveled extensively around the world. Family pets included a cat and a dog. He resides in Tel Aviv during the weekdays, and spends weekends at his home in the country, where he is an avid amateur gardener. One week prior to admission, the patient had sustained an upper respiratory tract infection for which he did not seek medical advice. He now presented to the emergency department (ED) with sudden-onset confusion and left-hand apraxia. He was initially suspected of having an ischemic stroke based upon non-contrast computed tomography (CT) performed the ED that showed a right parietal hypodense area (Fig. 1), and upon CT perfusion maps that showed a decrease in cerebral blood flow without a decrease in cerebral blood volume in the right middle cerebral artery territory. CT angiography was interpreted as being normal. The National Institutes of Health Stroke Scale (NIHSS) score was 4. The patient was admitted to our neurology department, and treatment for acute ischemic cerebral stroke consisting of clopidogrel, aspirin, and high-dose statins was started. Notably the patient did not have leukocytosis, and his C-reactive protein (CRP) levels were normal.

**Fig. 2:**
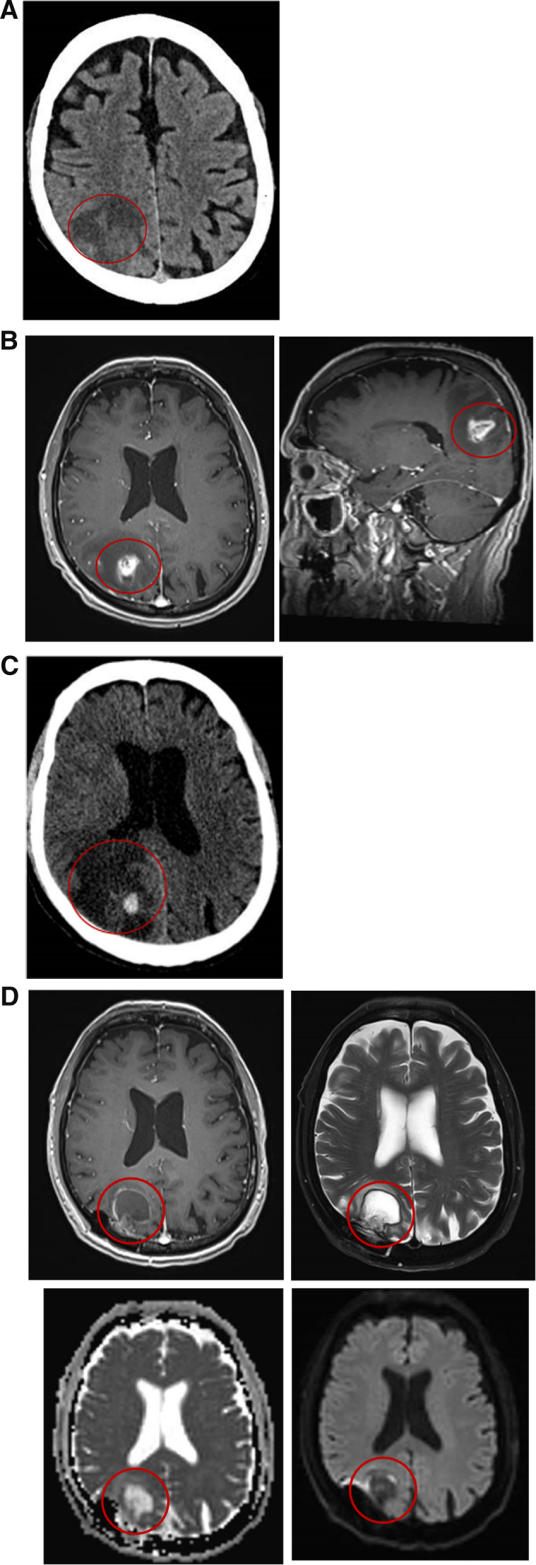
**A** Non-contrast CT scan showing the progress of the hypodense area in Fig. 1. **B** Magnetic resonance image (MRI) showing a right parietal space-occupying lesion (SOL), enhanced, surrounded by edema, with restricted diffusion in the periphery and no suspicion of bleeding on susceptibility weighted imaging. **C** A CT scan showing new hemorrhage in the SOL area. **D**  MRI axial view: Upper – post-contrast gadolinium T1WI (left) and T2WI (right) showing postoperative changes and significantly decreased edema and mass effect around the abscess. Lower – diffusion-weighted imaging/apparent diffusion coefficient showing thinner restricted diffusion on the periphery

**Fig. 3:**
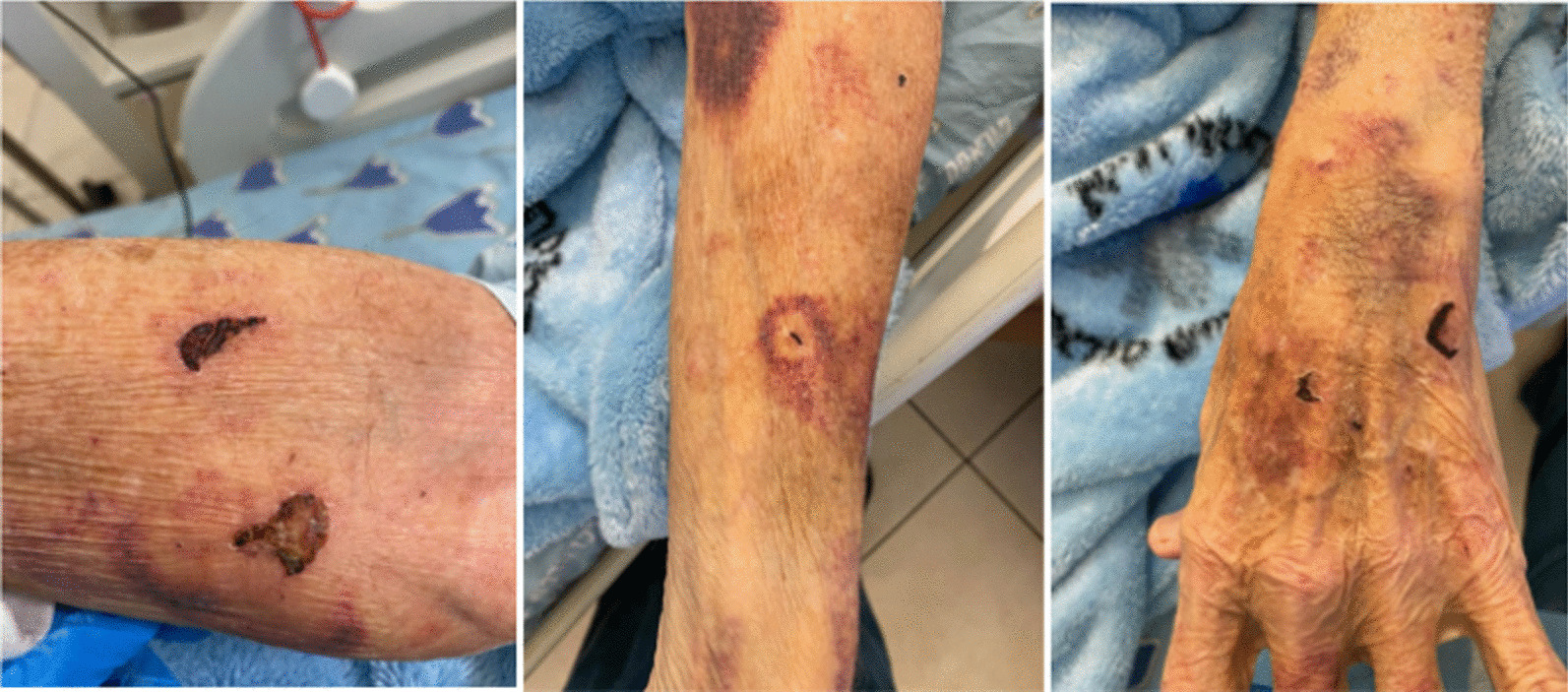
Multiple skin lacerations and cutaneous hematomas at different stages of evolution on both upper limbs which the patient and his spouse attributed to his gardening hobby

His echocardiogram, electrocardiogram, and electroencephalogram were interpreted as being normal. A carotid ultrasonography demonstrated right internal carotid artery stenosis of 60–70%. He was discharged home on dual-antiplatelet therapy 5 days after admission, with full resolution of the neurologic deficits and instructions to return for an elective right internal carotid end-antrectomy a week later. One day after discharge, however, the patient returned to the ED with recurrent confusion, left-hand apraxia, and new left hemianopsia. A non-contrast CT scan showed progression of the hypodense area (Fig. 2A). He was treated with an anti-epileptic drug based upon the differential diagnosis of a seizure episode. The increased vasogenic edema raised suspicion for a brain tumor, and a magnetic resonance imaging study showed a right parietal space-occupying lesion (SOL), enhanced, surrounded by edema, with restricted diffusion in the periphery, and no suspicion of bleeding on susceptibility weighted imaging (SWI) (Fig. 2B). The working diagnosis at this point was primary or secondary cerebral intra-axial tumor. The patient was transferred to the neurosurgical department for further evaluation. His physical examination was remarkable for left-sided proprioception impairment, multiple skin lacerations, and cutaneous hematomas at different stages of evolution on both upper limbs (the patient and his spouse attributed the skin lesions to his gardening activities) (Fig. 3). There were no obvious signs of skin infection. Intravenous high-dose dexamethasone was started. A right parietal craniotomy for resection of the lesion was scheduled. A few hours before the surgery, the patient underwent rapid neurological deterioration. He complained of severe headache, and an examination revealed new-onset severe (1/5) left-sided hemiparesis and right homonymous hemianopsia. He became stuporous shortly thereafter. The repeat CT showed new hemorrhage in the site of the lesion (Fig. 2C). He underwent an urgent right-sided parietal craniotomy. The Neuronavigational system (VectorVision, Brainlab, Munich, Germany) and ultrasonography were used to plan the extent of the craniotomy and the surgical approach. Electrophysiological mapping and monitoring included the minimal electrical current needed to elicit motor responses recorded from electromyography electrodes for detection of the motor cortex (direct cortical motor evoked potential threshold) or for estimation of proximity to the subcortical pyramidal structures (subcortical motor evoked potential threshold). The consistency of the excised lesion resembled a mixture of necrotic tissue with hematoma. The patient’s clinical progression, his medical history, and the intraoperative finding had raised no suspicion of an abscess, and the surgical specimens were sent only to pathology. The histopathology result was of an abscess with surrounding fibrovascular tissue organization, consistent with a pyogenic abscess. Neither a gram stain nor an acid-fast stain were done. A peripherally inserted central catheter line was installed and empiric antibiotic therapy consisting of ceftriaxone 2gr*1/d and metronidazole 500 g*3/d was initiated. This antibiotic regimen was given to the patient as an empiric antibiotic treatment due to a pathological report of a brain abscess without any cultures taken. Most single brain abscesses occur via direct spread from a contiguous site, such as otitis media, mastoiditis, sinusitis and dental infections and empiric antibiotic treatment is given accordingly. Nocardia was not part of the differential diagnosis at that time.

The surgical specimen underwent “de-paraffinization” and was sent for broad range16S bacterial and 18 S fungal polymerase chain reaction. A positive signal was obtained with primers targeting the mycobacterial gene heat-shock protein 65. Further sequencing of this amplicon revealed a match with Nocardia cyriacigeorgica. The antibiotic therapy was changed empirically to meropenem 2gr*3/d and trimethoprim\sulfamethoxazole 240 mg (trimethoprim)*3/d. The patient recovered well from the surgery, and all of the neurological symptoms did not reoccur. He was discharged home at postoperative day 14 and continued on intravenous antibiotic therapy as an outpatient, for a total duration of 6-weeks. The patient returned for follow-up two months post-surgery. He reported feeling well for the most part, and was free of neurological symptoms and of significant treatment side effects. A follow-up brain imaging study was done at that visit and revealed improvement, with less edema, less enhancement and restriction (Fig. 2D).

## Discussion and conclusions

Nocardia species have been called “great imitators” because of their variety of clinical presentations [22]. Nocardia is a well-known mimicker of several conditions of brain involvement, including primary brain tumor, metastases, and ischemic stroke [8, 23–25]. Nocardiosis is most commonly an opportunistic infection that causes both localized and systemic infections in the immunocompromised population. Brain involvement is rare, with N. cyriacigeorgica accounting for 2% of all brain abscesses [26]. A Nocardial brain abscess is 2.5 times more common among men [3]. The mortality rate of a Nocardia brain abscess is greater than 50%, which is three times higher than that of other bacterial causes of cerebral abscesses [10]. A Nocardial brain abscess is rare among the immunocompetent population. Publications consist solely of case reports involving one to three patients, and all of them were caused by species other than N. cyriacigeorgica, including N. farcinica, N. otitidiscaviarum, N. brasiliensis and N. abscessus [10–16, 27].

N. cyriacigeorgica was first isolated in 2001 by Yasin et al. from a patient with chronic bronchitis [17]. It has since been isolated from different clinical specimens and with a vast geographical distribution, including the USA, western Europe, Greece, Turkey, Japan, Thailand, and Canada [1]. A few case reports have described N. cyriacigeorgica brain abscess in immunocompromised patients [6–8, 17, 18, 28]. The main immunocompromised states that cause predisposition to brain abscesses are AIDS infection and diabetes mellitus [3]. Infections from N. cyriacigeorgica among immunocompetent patients are rare, and our search of the literature yielded case reports limited only to non-central nervous system infections [9].

It is well known that glucocorticoids have dose-dependent inhibitory effects on a broad range of immune system functions [19]. These inhibitory affects probably start at a low dose of glucocorticoid use [19]. However, the effects on phagocytic cell function with long-term, low-dose use are usually minimal [19]. The dose required to induce an increased risk of serious infections is a matter of controversy, with some studies claiming that a low dose of corticosteroids confers an increased risk while other studies argue that an increased risk occurs only with higher doses [20]. While the immunocompromising effects of the low dose prednisone were unknown in our patient, the fact that he was on long-term, low dose steroids, certainly possesses an important risk factor.

Nocardia species, as an opportunistic source of infection, commonly manifest in immunocompromised hosts, specifically in patients with conditions that impair T cell-mediated immunity [21]. These patients commonly receive prolonged regimens of glucocorticoids in addition to other immunosuppressant drugs or have other underlying immunosuppressive conditions [19].

The clinical course of Nocardiosis can be misleading, which was reflected in the current case whose presentation was initially thought to be an ischemic stroke and later determined as being a cerebral lesion. It can sometimes be difficult to recognize the source of symptoms as being an abscess at presentation, and a high clinical suspicion is needed. Frequent skin injuries incurred during gardening were a likely source of Nocardia inoculation, and, in retrospect, together with his long-term prednisone consumption history, were an important diagnostic clue in our patient.

In conclusion, Nocardia species are rare causes of brain abscess in immunocompetent patients. Their clinical presentation can mimic other, more common cerebral diseases. We report a case of a brain abscess caused by N. cyriacigeorgica in an immunocompetent patient that was thought to be an ischemic stroke at presentation and a space-occupying lesion later on. We believe that frequent skin injuries during gardening were a likely source of his Nocardia inoculation.

## Data Availability

The data used to support the findings of this study are available from the corresponding author upon request.

## Abbreviations

N. cyriacigeorgica: Nocardia cyriacigeorgica
ED: Emergency department
CT: Computed tomography
SOL: Space-occupying lesion
SWI: Susceptibility weighted imaging
AIDS: Acquired immunodeficiency syndrome
